# How to measure children’s feet: 3D foot scanning compared with established 2D manual or digital methods

**DOI:** 10.1186/s13047-023-00618-y

**Published:** 2023-04-15

**Authors:** Juliane Mueller, Monika Richter, Kathrin Schaefer, Jonathan Ganz, Jörg Lohscheller, Steffen Mueller

**Affiliations:** 1 Department of Computer Science, Therapy Sciences, Trier University of Applied Sciences, Trier, Germany; 2 Prüf- und Forschungsinstitut Pirmasens e.V, Department of Shoe Technical Research and Development, PFI Germany, Pirmasens, Germany; 3 AImotion Bavaria, Technische Hochschule Ingolstadt, Ingolstadt, Germany; 4 Department of Computer Science, Trier University of Applied Sciences, Trier, Germany

**Keywords:** Foot length, Foot width, Shoe size, Shoe industry

## Abstract

**Background:**

In infants and young children, a wide heterogeneity of foot shape is typical. Therefore, children, who are additionally influenced by rapid growth and maturation, are a very special cohort for foot measurements and the footwear industry. The importance of foot measurements for footwear fit, design, as well as clinical applications has been sufficiently described. New measurement techniques (3D foot scanning) allow the assessment of the individual foot shape. However, the validity in comparison to conventional methods remains unclear. Therefore, the purpose of this study was to compare 3D foot scanning with two established measurement methods (2D digital scanning/manual foot measurements).

**Methods:**

Two hundred seventy seven children (125 m / 152 f; mean ± SD: 8.0 ± 1.5yrs; 130.2 ± 10.7cm; 28.0 ± 7.3kg) were included into the study. After collection of basic data (sex, age (yrs), body height (cm), body weight (kg)) geometry of the right foot was measured in static condition (stance) with three different measurement systems (fixed order): manual foot measurement, 2D foot scanning (2D desk scanner) and 3D foot scanning (hand-held 3D scanner). Main outcomes were foot length, foot width (projected; anatomical; instep), heel width and anatomical foot ball breadth. Analysis of variances for dependent samples was applied to test for differences between foot measurement methods (Post-hoc analysis: Tukey-Kramer-Test; α=0.05).

**Results:**

Significant differences were found for all outcome measures comparing the three methods (*p*<0.0001). The span of foot length differences ranged from 3 to 6mm with 2D scans showing the smallest and 3D scans the largest deviations. Foot width measurements in comparison of 3D and 2D scans showed consistently higher values for 3D measurements with the differences ranging from 1mm to 3mm.

**Conclusions:**

The findings suggests that when comparing foot data, it is important to consider the differences caused by new measurement methods. Differences of about 0.6cm are relevant when measuring foot length, as this is the difference of a complete shoe size (Parisian point). Hence, correction factors may be required to compare the results of different measurements appropriately. The presented results may have relevance in the field of ergonomics (shoe industry) as well as clinical practice.

## Introduction

Compared to adult feet, children's feet have characteristic differences in their structure and function [1, 2]. One characteristic is the pediatric fat pad in the midfoot in children, which protects against excessive pressure until the musculoskeletal system has adapted to an upright gait [3]. This initially leads to great flexibility of the child's foot. Foot geometry in children changes rapidly during growth and maturation and depend on the child’s age of beginning to stand and walk [1, 4, 5]. Therefore, a wide heterogeneity of foot shape is typical in infants and young children. However, this is not necessarily associated with pathological deformities [6]. In detail, foot size (length, width) enlarges with increasing age of children [1, 4, 6]. During child development, the foot grows predominantly in length and less in width [1]. Therefore, it has been shown that the relationship between foot width and length evolves towards a narrower foot during growth [1]. Thus, until the age of 8 years, children’s feet have a wider shape compared to older ones. When children become older than 8 years the proportion of their feet becomes more and more similar to those of adults [1]. Furthermore, the longitudinal arch of the feet declines until the age of six and remains on a constant level afterwards [1]. One of the most basic and commonly used parameters, among others, for measuring children's feet is foot length [1, 5]. In particular, foot length serves as a basic quantity to normalize further foot parameters to account for growth and development of the aging child [1, 5]. Due to the great heterogeneity of foot shape as well as rapid growth and maturation, children represent a very special group for foot measurements and the footwear industry.

The importance of foot measurement for footwear fit and design, as well as clinical applications is evident [1, 7–14]. Although knowledge of the high heterogeneity in foot shape in children is evident [1, 4, 6, 13, 14], the footwear industry still bases the last as well as the shoe development predominantly just on the foot length and ball width [15]. However, it is obvious that children can have the same foot length, but different foot shapes (e.g. wide versus narrow) [4, 6, 15]. This fosters a mismatch between the manifold foot and shoe shapes [1, 4, 15].

Nowadays, manual foot measurements (length/ forefoot width) by use of assistant devices (e.g. german WMS® foot measurement system for children) are the established gold standard in the shoe stores. However, these measurements do not consider the individual and multidimensional foot shape. To enable a more individualized foot analysis, 2D foot scans are only used in special cases, e.g. assignment of individual sport shoes for athletes, and are not available to the general public. Nevertheless, 2D foot scans are limited and cannot measure vertical dimensions (e.g. navicular height or volume/girth) [6]. This issue can be solved by three-dimensional measurements that provide a detailed scanning of the foot shape in all spatial dimensions [4, 7, 15]. Nowadays, there are three-dimensional foot measurement technologies that allow rapid measurement and data evaluation, including recommendation for matching shoes in the shoe store [7]. However, this is limited to selected shoe stores with access to these measurement technologies and is mainly used for adults [7].Therefore, the shoe industry has the need to implement three-dimensional foot data for fit determination already at the stage of shoe-last production [12, 15]. In recent years, the development of technologies (3D scanners, computer-aided design (CAD), computer-aided manufacturing (CAM)) has enabled the production of lasts based on three-dimensional foot data [12, 15].

Nevertheless, information on validity and comparability of the two and three-dimensional measurement methods (manual, 2D, 3D) is not clear yet. Therefore, the purpose of this study was to compare 3D foot scanning with two established measurement methods (2D scanning and 2D manual foot measurements).

## Material and methods

### Participants

The study was approved by the institution’s Ethical Committee of the University of Potsdam (No. 04/2018). Children were recruited from local primary schools as well as local children shoe stores. Children’s parents were informed about the purpose and applied methods and gave informed consent before children’s voluntarily participation in the study. Based on inclusion (age 5-10 yrs) and exclusion criteria (no acute/chronic pain and/or injury at the locomotor system), measurements were performed on *n =* 297 children. For final analysis *n =* 277 children (125 m / 152 f; mean ± SD: 8.0 ± 1.5yrs; 130.2 ± 10.7cm; 28.0 ± 7.3kg) could be considered since 20 presented incomplete data sets (17 missing values for 3D scans; 3 missing values for 2D scans) and were therefore excluded. Table 1 shows detailed information (including mean and 95% confidence interval) on anthropometric data of the included participants.

**Table 1:** Anthropometric data of included participants (mean; 95% Confidence interval (CI); range)

**Number of participants**	**Gender (female / male)**	**Age (yrs)**			**Body height (cm)**			**Body weight (kg)**		
		**Mean (Range)**	**Upper 95%-CI**	**Lower 95%-CI**	**Mean** **(Range)**	**Upper 95% CI**	**Lower 95% CI**	**Mean** **(Range)**	**Upper 95% CI**	**Lower 95% CI**
277	152 / 125	8.02 (5.05 – 10.96)	8.20	7.85	130.17 (105.50 -167.00)	131.44	128.90	28.04 (15.80 – 62.10)	28.91	27.18

### Experimental protocol

After parents’ informed consent to the study was given, information on age and sex were collected as well as anthropometric measurements (body height (cm) measured with seca mobile stadiometer 217; body weight (kg) measured with seca flat scale 899, seca Germany) were conducted barefoot while participants remained wearing all-day clothes. Afterwards, geometry of the right foot was measured in static condition (stance) with three different measurement systems (fixed order): (1) manual foot measurement [1], (2) 2D foot scanning on a 2D desk scanner and (3) 3D foot scanning by use of a 3D hand-held scanner [6]. All foot measurements were performed in a bipedal stance. The same experienced examiner and two scientific assistants performed data acquisition throughout the whole study. The whole measurement procedure took about ten minutes per child.

### Manual Foot Measurements (MF)

**Fig. 1:**
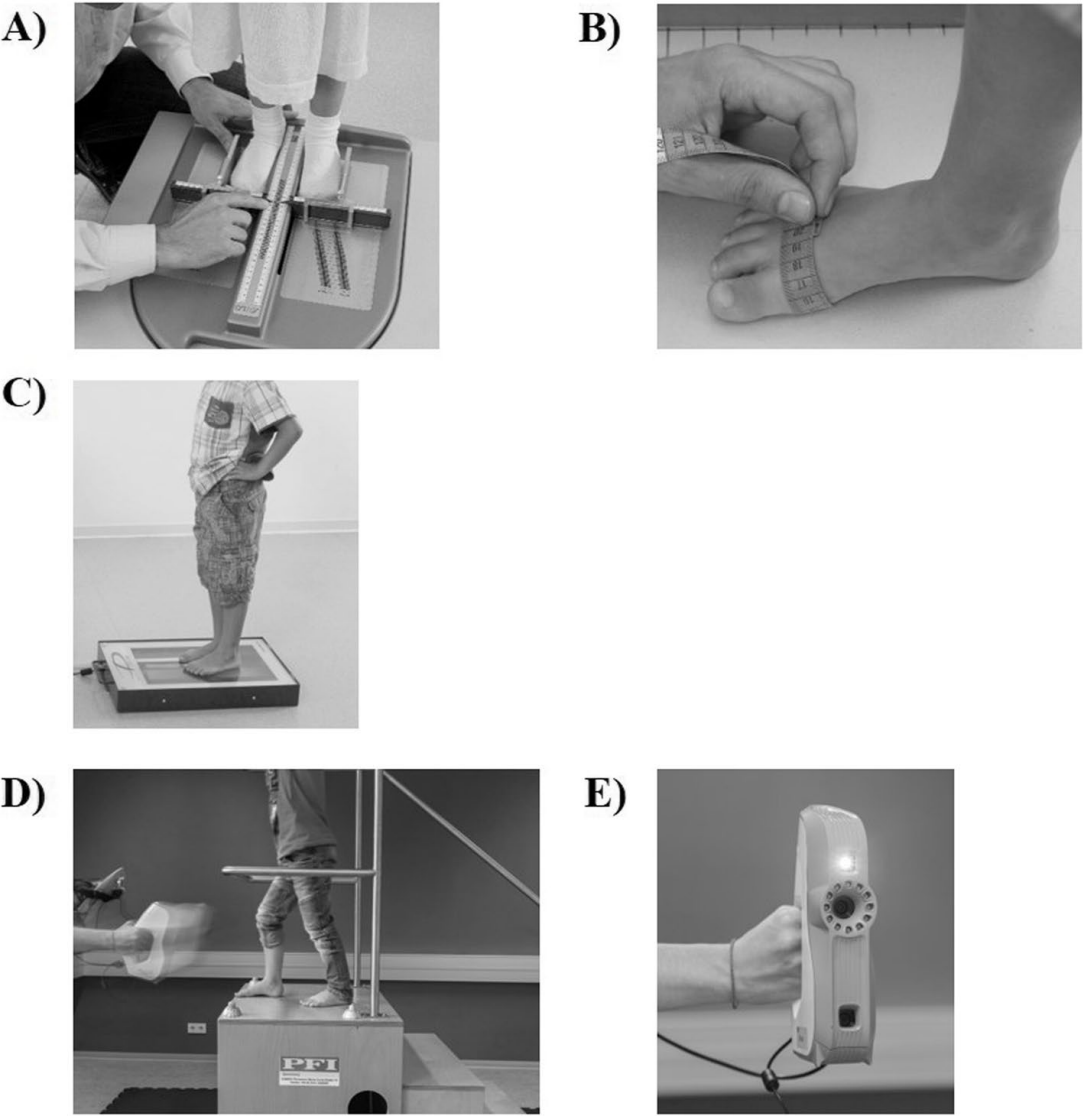
Devices and setups for the Manual Foot Measurements (A/B), 2D Foot Scan (C) and 3D Foot Scan (D/E). **A** standardized foot measuring device (WMS® foot measurement system, DSI, Offenbach, Germany). **B** setup for manual foot ball breadth measurement. **C** 2D desk scanner (iScan 2D, IETEC Biomechanical Solutions, Germany)). **D** 3D foot scan measurement setup. **E** 3D hand-held light scanner (Artec Eva; Artec Group, Luxembourg)

Static foot length (FL; [mm]) and (projected) fore foot width (FW; [mm]) were measured in a standing position with a standardized foot measuring device developed to determine shoe size (WMS® foot measurement system with an attached millimeter scale, DSI, Offenbach, Germany; Fig. 1A) [1, 16]. The device consists of a base plate, a rear margin, and a separator (left/right foot) orthogonal to the rear margin. The feet were set left/right to the separator having contact on the medial foot side and with the heel all the way back to the rear margin. Foot length and width are measured with a front and side slider (orthogonal to separator/rear). Therefore, foot length is defined as maximum heel to longest toe distance and foot width is the maximum forefoot width. In addition, the anatomic foot ball breadth (Fig. 1B) was measured with a tapeline [mm] around the big toe joint and the small toe joint in a standing position with half-body weight bearing on each foot (Fig. 1B). All outcomes are detailed in Table 2.

**Table 2:** Outcome measures for all three measurement methods

**Dimensions**	**Outcomes** **(cm)**	**Definitions**		**Methods**		
				**Manual foot measurement**	**2D foot scan**	**3D foot scan**
**Length**	Foot length, FL	The direct distance from maximum heel point to the most anterior point of the longest toe (first or second).		X	X	X
**Width**	Projected foot width, FW_P	The maximum horizontal forefoot distance between metatarsal tibial to metatarsal fibular		X	X	
	Anatomic foot width, FW_A	The maximum diagonal distance between metatarsal tibial to metatarsal fibular measured at XX	Fig. 2		X	X
	(technical) Instep width (at 50% foot length), FW_50	The maximum horizontal distance between metatarsal tibial to metatarsal fibular measured at 50% foot length	Fig. 2		X	X
	(technical) Heel width (at 20% foot length), HW	The maximum width straight from the medial to the lateral side measured at 20% foot length	Fig. 2		X	X
**breadth**	Anatomical foot ball breadth, FB	The breadth measured around the big toe joint and the small toe joint.	Fig. 1B/2	X		X

### 2D Foot Scan (2D)

To scan the children’s right foot, the participants were instructed to stand still on a 2D desk scanner (iScan 2D, IETEC Biomechanical Solutions, Germany; Fig. 1C) in a hip wide bipedal stance with weight equally distributed across both feet. Shoes and socks were removed before the measurements. During the scan procedure, a lightproof blanket was placed over the feet and the scan bed to ensure optimal light conditions for the scanning procedure. Afterwards, the examiner visually inspects the scanning result. In case of reduced scan quality (e.g. foot placed too close to the boarders of the scan platform; blurry image due to movement artefacts), the scanning procedure was repeated. The foot scans were recorded within A3 size and a resolution of 96 dpi (= 1 Dot per Inch = 25.4 mm).

**Fig. 2:**
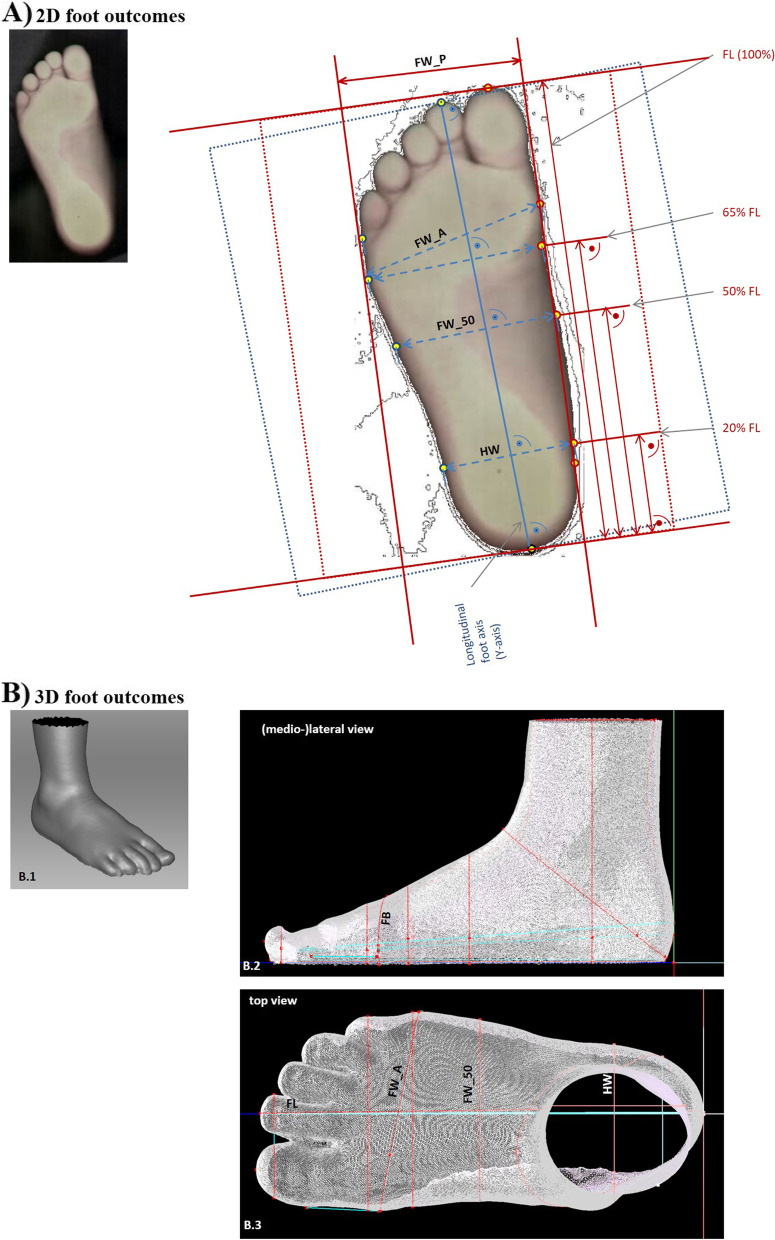
Visualization of all outcomes of the 2D and 3D Foot Scan. **A** 2D Foot Scan Outcomes. **B** 3D Foot Scan Outcomes

*Image processing:* Each foot scan was analyzed using a custom-made image recognition software (FeetAnalyzer, MATLAB 2018). This software allowed a semi-automatic detection of the landmarks for the calculation of the main outcome measures. Every automatic detection was controlled through visual inspection by an experienced examiner. If automatic detection failed (e.g. due to movement artefact), the investigator applied manual correction (<2% of all cases analyzed). Main outcome measures for 2D scans are foot length (FL; [mm]) and five parameters of foot width: projected (fore)foot width (FW_P, [mm]), anatomic (fore)foot width (FW_A), (technical) foot ball width at 65% foot length (FW_65, [mm]), (technical) foot instep width at 50% foot length (FW_50; [mm]) and (technical) heel width (HW; [mm]) at 20% foot length. All outcomes are detailed in Table 2 and visualized in Fig. 2.

### 3D Foot Scan (3D)

Three-dimensional foot scans were executed on a platform (height: 120 cm) bordered by a handrail and accessible by a stair. Children stood still in a slightly lunge position with the right foot in the front and at the edge of the platform (Fig. 1D). Children were instructed to stand still and look straight forward (not downward) with hands fixing the handrail. During measurement, an experienced examiner scanned the right foot by driving the hand-held 3D light scanner (Artec EVA, Artec Group, Luxembourg) [6, 17] around the foot beginning at the lateral heel side over the toes leading to the medial heal side. In case of foot movement artefacts, the measurement was repeated. Scanning was performed at a speed of 16 frames per seconds and the depth of the scanning field was adjusted to 400 mm and 100 mm. The 3D accuracy of the scanner is up to 0.05 mm, and the 3D resolution is up to 0.1 mm. During scanning, a laptop with Artec EVA Studio software (version 9.2.3.15; Artec Group) was used to host the 3D scanner.

*Image processing:* The 3D scan files were processed with an industry-acclaimed software package for advanced 3D scanning and data processing (ARTEC Studio V11; Artec Group, Luxembourg). For each scan file a 3D model of the foot was created including the following steps: visual inspection of the scans to check for possible scan errors (e.g., cramped or lifted toes, defective objects in the scan area, noise etc.), scan alignment, global data registration, fusion of data into a 3D model (Fusion), and final editing of the 3D model (e.g. cut to size). Thereafter, each scan was oriented manually according to the established coordinate system (y-axis = longitudinal foot axis from heel to second toe). The origin of the coordinate system was set to the maximum arch point of the heel. Afterwards the model was exported to STL format. Subsequently, the STL file was imported into a custom-made analysis software for 3D foot model (FootOpenGL, PFI, Germany). To obtain the information for the desired main outcome measures, defined measurement points, cuttings and lines are added to the model (Fig. 2). The used software consisted of an automatic detection mode for the points and cuttings for the defined main outcomes measures. Every automatic detection was controlled through visual inspection by an experienced examiner. If automatic detection failed, the investigator applied manual correction (<2% of all cases analysed). Main outcome measures for 3D scans are foot length (FL; mm]), foot ball breadth (FB; [mm]) and four parameters of foot width: projected (fore)foot width (FW_P; [mm]), anatomic (fore)foot width (FW_A), (technical) foot instep width at 50% foot length (FW_50; [mm]) and (technical) heel width (HW; [mm]) at 20% foot length. All outcomes are listed in Table 2 and visualized in Fig. 2.

### Data analysis and statistics

Initially, data was recorded in a case report form (CRF) followed by transforming and saving in a statistical database (JMP Statistical Software Package 14, SAS Institute®). All measures were checked for plausibility (e.g. range check, age: 5-10 years; body height: < 2.00 m; body weight: < 120 kg). Implausible values were recalculated or corrected as documented in the handwritten CRF. Otherwise values were erased out of the database. Statistical analysis was performed descriptively calculating mean and standard deviation followed by inferential statistics. All outcomes were checked for normal distribution with Shapiro-Wilk-Test. Following this, analysis of variance (e.g. one-way repeated-measures ANOVA; paired t-test) for dependent samples was applied to test for differences between measurement methods. In case of significance, Tukey-Kramer-Test was applied for post-hoc analysis. Level of significance was set to α = 0.05. To account for multiple testing, the level of significance was adjusted (Bonferroni correction) to α = 0.008. In addition, Bland and Altman analysis including bias and 95%-limits of agreement (LoA) were calculated to evaluate reproducibility between the methods used (manual vs. 3D; manual vs. 2D; 2D vs. 3D) [18, 19]. Further, heteroscedasticity was analyzed calculating Pearson correlation (for the mean value of two measurement approaches and the difference between the two measurement approaches).

## Results

Results for all outcomes, including mean ± SD as well as ANOVA-analysis, are reported in Table 3. Significant differences were found for all outcome measures comparing the three methods (*p* < 0.0001). Differences ([mm]) between the methods are reported in Table 3.

**Table 3:** Results for all outcome measures for all three foot measurement methods (mean ± SD and analysis of variances) and differences between foot measurement methods for all outcome measures

**Dimension**	**Outcomes**	**Methods**			*p*-value*	**Differences between methods**		
		**Manual foot measurement (mm)**	**2D Foot Scan (mm)**	**3D Foot Scan (mm)**		**Manual foot measurement vs. 2D (mm)**	**Manual foot measurement vs. 3D (mm)**	**2D vs. 3D (mm)**
**length**	Foot length, FL	201.4 ± 18.0	197.5 ± 17.4	203.7 ± 18.1	<0.0001	+3.9	-2.3	-6.2
**width**	Projected foot width, FW_P	74.9 ± 6.0	76.3 ± 6.8	- -	<0.0001	-1.4	- -	- -
	Anatomic foot width, FW_A	- -	78.0 ± 6.8	80.7 ± 6.9	<0.0001	- -	- -	-2.7
	(technical) Instep width (at 50% foot length), FW_50	- -	67.7 ± 6.0	68.6 ± 6.0	<0.0001	- -	- -	-0.9
	(technical) Heel width (at 20% foot length), HW	- -	51.0 ± 4.6	53.5 ± 4.5	<0.0001	- -	- -	-2.5
**breadth**	Anatomical foot ball breadth, FB	200.2 ± 17.5	- -	197.0 ± 16.6	<0.0001	- -	+ 3.2	- -

Regarding foot length, differences ranged from 3 mm to 6 mm with 2D scans showing the smallest and 3D scans the largest values.

Foot ball breadth measurements showed a difference of 3 mm between MF and 3D scans.

Foot width measurements in comparison of 3D and 2D scans always showed higher values for 3D measurements with the differences ranging from 1 mm to 3 mm.

The results of bias and limit of agreement analysis as well as heteroscedasticity (person correlation) for comparison of manual, 2D and/or 3D measurements for selected parameters of foot length, width and breadth are detailed in Table 4.

**Table 4:** Indicators^a^ of bias and heteroscedasticity for comparison of manual, 2D and 3D measurements for parameters of foot length, width and breadth

**(A) Manual foot measurement (MF) vs. 2D Foot Scan (2D)**						
**outcome**	**MF** [mm]	**2D** [mm]	**Pearson R**	**Bias**	**Upper LoA**	**Lower LoA**
				[mm]	[mm]	[mm]
Foot length, FL	201.4 ± 18.0	197.5 ± 17.4	0.14	0.40	1.33	-0.54
Projected foot width, FW_P	74.9 ± 6.0	76.3 ± 6.8	-0.05	-0.13	0.39	-0.66
**(B) Manual foot measurement (MF) vs. 3D Foot Scan (3D)**						
**outcome**	**MF** [mm]	**3D** [mm]	**Pearson R**	**Bias**	**Upper LoA**	**Lower LoA**
				[mm]	[mm]	[mm]
Foot length, FL	201.4 ± 18.0	203.7 ± 18.1	-0.02	-0.23	0.26	-0.72
Anatomical foot ball breadth, FB	200.2 ± 17.5	197.0 ± 16.6	0.11	0.32	2.01	-1.38
**(C) 2D vs. 3D Foot Scan (3D)**						
**outcome**	**2D**	**3D**	**Pearson R**	**Bias**	**Upper LoA**	**Lower LoA**
				[mm]	[mm]	[mm]
Foot length, FL	197.5 ± 17.4	203.7 ± 18.1	-0.14	-0.63	0.34	-1.59
Anatomic foot width, FW_A	78.0 ± 6.8	80.7 ± 6.9	-0.06	-0.27	0.10	-0.64

^a^Bias [mm]; 95%-Limits of Agreement (LoA;[mm]); Pearson correlation

## Discussion

The purpose of this study was to compare 3D foot scanning to the established methods of manual foot measurements (2D) and 2D foot scanning in primary school childrens´ feet. The main result is that there are significant differences for all outcomes of foot length and width comparing the three methods.

The study results show that the different methods somewhat under- and/or overestimate the single outcomes analyzed. This is in accordance to previously reported studies [9]. The presented results show that there is no relevant/significant heteroskedastic error between measurement methods that could have arisen due to variance in foot size in our population. Therefore, smaller feet are not expected to have a smaller measurement difference between the different methods compared to larger feet. Therefore, there is no bias in the average error estimations. Mainly the differences between the measurement approaches are due to the nature of the measurement methods themselves. In detail, all foot dimensions collected with the 3D scanner were greater compared to the 2D scanning as well as the manual foot measurements. One reason for this could be that the 3D scanner detects the outermost points of the superficial boundaries (e.g. metatarsal head) more precise than the manual foot measurements as well as the 2D scan when measuring foot length, forefoot width and heel width [9]. Another reason may be that the examiner presses the soft tissue surrounding the measuring points with the material of the slide during the manual foot measurement [9]. This can lead to a measurement error resulting e.g. in smaller values ​​in foot length and/or width. Moreover, the foot measures collected from the 2D scanning were smaller than those collected using the manual measurement methods as well as the 3D scanning. One reason for this, that needs to be discussed, might be the shape of the human foot: It is curved upwards at the outer (medial, lateral) edges and does not lie completely flat with the entire plantar surface. Because of this, the footprint on the scanner board might be reduced at the edge of the foot, and the foot scan contour captured tends to be smaller than the actual plantar surface contour [9]. Consequently, a standardized measurement procedure is desirable as well as an adequate training for the examiner should be carried out before the use/application of the described measurement methods.

Moreover, the differences between all outcomes of the three analyzed measurement methods are statistically significant but the clinically or ergonomically relevance must also be questioned [7, 9]. Differences of about 0.6 cm are important for the foot length as this is the differences of one complete shoe sizes in accordance with the Parisian point [3]. Furthermore, even half sizes are relevant in some shoe size systems (e.g. US) therefore even the difference between 3D and manual measurements seems of ergonomic relevance. As this is the case for all methods for foot length, a correction procedure (e.g. application of a corrective factor) has to be discussed [20].

Regarding foot width, the differences, presented results ranged between 1 mm to 3 mm, are ergonomically of relevance as the foot width in the german WMS®-system for children’s feet/shoes (wide – middle – small children feet) clusters the foot width by adding 18 mm for each width category.

The 3D foot scanning is the technological gold standard for the assessment of foot morphology [9, 12, 13, 21]. The advantages of using the 3D scanning system to collect foot measures is the high precision and accuracy of the different systems [17]. The 3D foot scanning allows the assessment of volumetric and surface data and provides more detailed information on foot size as well as foot shape in all dimensions compared to the manual measurement as well as the 2D scanning [9]. This is especially important for the growing foot of the children [4]. Nevertheless, the high initial set-up cost as well as the time needed for the processing of the data (about 1 to 2 hours for each scan) are disadvantages to be named. Moreover, the practical suitability largely differs between the different 3D systems. The one used within the presented study needs more time for a scan (up to 5 min) compared to the manual foot measurement (1 to 2 min). Besides, the 3D measurements can be significantly accelerated by using a stationary camera measurement system placed around the foot instead of the hand-held mobile scanner.

Based on the presented results, our study supports the use of 3D foot scanning measurement for collecting foot anthropometric data in school children aged five to ten years of age, especially as a basis for collecting detailed information on foot shape and size in all three dimensions for the last construction of children shoes. A purely individualized shoe production based on 3D scan data in the context of children's feet should not be the goal (high shoe costs), therefore the development of 3D data based shoe lasts for shoe production for these age groups should be aspired. Besides, using different devices/techniques to measure foot measures may produce inconsistent results between studies. Therefore, it is important to consider the measurement method differences when comparing foot data between studies [9, 21].

Certain limitations have to be considered when interpreting these results. In addition, not all outcomes are available for all measurement methods at a time based on the nature of the measurement method (e.g. 2D scanning does not allow measuring the foot girth). In this case, only the two available methods were used for comparison to allow a more detailed comparison of outcome measures that are not only based on foot length and forefoot width. Information on accuracy and reliability of the measuring methods are not analyzed within this study. However, reliability and validity of the measurement methods is described elsewhere and evaluated as good to excellent [1, 9]. The different baseline positions for the foot measurements (2D manual/digital: two-legged parallel stance vs. 3D: slightly lunge) may have influenced the results due to the possible differences in weight distribution on the feet. The slight lunge was technically necessary in order to be able to perform the three-dimensional scan properly, without foreign bodies in the scan area. In order to keep this effect as low as possible, the investigator gave the children specific instructions on how to assume the slight lunge position in order to distribute the weight on both legs as much as possible. However, an influence cannot be completely ruled out.

## Conclusion

The presented data may be relevant in the field of ergonomics (shoe industry) as well as clinical practice. For application, it shows the importance, that the measurement method for the feet should be in line with the measurements method of the shoe/last. In addition, the finding of the presented study suggests that when comparing foot data among different studies, it is important to consider the differences caused by the applied measurement methods. Based on the presented (three-dimensional) data, a foot typing might be advantageous for further development of children shoe lasts that account for a higher number of foot shape variability in children.

## Data Availability

The datasets used and/or analysed during the current study are available from the corresponding author on reasonable request.

## Abbreviations

2D: Two-dimensional
3D: Three-dimensional
FB: Foot ball breadth
FL: Foot length
FW_A: Anatomic (fore)foot width
FW: Fore foot width
FW_P: Projected fore foot width
FW_50: (Technical) foot instep width at 50% foot length
HW: (Technical) heel width at 20% foot length
MF: Manual Foot Measurements
